# 726 Evaluating Outcomes and Pharmacoeconomics Associated with Collagenase Use in Burn Patients

**DOI:** 10.1093/jbcr/irac012.280

**Published:** 2022-03-23

**Authors:** Allison N Boyd, Todd A Walroth, Madilyn Eberle, Cortni Grooms, Victoria Hull, Brett C Hartman

**Affiliations:** Eskenazi Health, Indianapolis, Indiana; Eskenazi Health, Indianapolis, Indiana; Purdue University College of Pharmacy, West Lafayette, Indiana; Richard M. Fairbanks Burn Center, Indianapolis, Indiana; Richard M. Fairbanks Burn Center, Indianapolis, Indiana; Richard M. Fairbanks Burn Center, Indianapolis, Indiana

## Abstract

**Introduction:**

Collagenase is routinely used for partial thickness burns at our health-system. A 30 gram tube costs over $300, which creates financial challenges for uninsured patients and impacts revenue capture. The objective of this medication utilization evaluation was to characterize the use of collagenase and evaluate compliance to our institution’s criteria for use.

**Methods:**

All admitted patients who received collagenase for burn injury from 3/14/21 to 6/14/21 were included. Patients were excluded if they were not admitted to the burn unit or left against medical advice. A cost analysis was also conducted.

**Results:**

A total of 26 patients were included with a mean (SD) age of 45 (17) years. Most burns were thermal (77%) with a median (IQR) of 7.5% (1.9,13.5) total body surface area (TBSA). Twenty-three patients presented with mixed partial thickness or partial thickness burns (89%). The three remaining patients had full thickness burns, which is not an indication for collagenase use at our institution. There was a median of 1 (0,1) collagenase treatment day per TBSA. Median length of hospital stay was 5 (2,22) days with a length of stay per TBSA of 1 (1,2). Approximately 58% of patients required a surgical procedure. Of these, 8 had documented graft loss or failure while 7 did not. In those who experienced graft loss, median TBSA was higher [31.4 (7.8,57.5) vs. 10 (2.5,15); p = 0.269] and they required more surgeries [7 (1,9) vs. 1 (1,3); p = 0.104]. A potential total revenue of $428,280 was found. Additional cost data are provided in Table 1.

**Conclusions:**

There was a high level of compliance with criteria for use, with some opportunities for improvement. Over 40% of patients who received collagenase for partial thickness burns were treated non-operatively, supporting its likely benefit, despite the cost. The potential exists for significant revenue for the health-system.

